# Utilization of Quinoa Post-Fermentation Waste as a Medium for Carotenoid Production by Yeast

**DOI:** 10.3390/molecules31020329

**Published:** 2026-01-18

**Authors:** Ewa Kulczyk-Małysa, Elżbieta Bogusławska-Wąs, Patrycja Jaroszek, Katarzyna Szkolnicka, Artur Rybarczyk

**Affiliations:** 1 Department of Applied Microbiology and Human Nutrition Physiology, Faculty of Food Sciences and Fisheries, West Pomeranian University of Technology in Szczecin, Papieża Pawła VI 3, 71-459 Szczecin, Poland; elzbieta.boguslawska-was@zut.edu.pl (E.B.-W.);; 2 Department of Toxicology, Dairy Technology and Food Storage, Food Science and Fisheries Faculty, West Pomeranian University of Technology in Szczecin, Papieża Pawła VI 3, 71-459 Szczecin, Poland; katarzyna.szkolnicka@zut.edu.pl; 3 Department of Animal Nutrition and Feed Science, Wrocław University of Environmental and Life Sciences, Chełmońskiego 38C, 51-630 Wrocław, Poland; artur.rybarczyk@upwr.edu.pl

**Keywords:** carotenoids, plant waste, *Rhodotorula* spp., *Sporobolomyces* spp., bioactive powder

## Abstract

Carotenoids are a diverse group of isoprenoid compounds found in nature. As natural pigments and bioactive compounds, carotenoids are used in various industries as functional additives. The increasing knowledge about the disadvantages of synthetic carotenoid production has drawn attention to the potential of carotenogenic yeasts and the use of food industry waste. This study analyzed the potential of post-fermentation waste from fermented quinoa production as a culture medium. For this purpose, reference yeast strains and strains isolated from various environments were used. The C:N ratio in the waste used was determined, and then the yeast was cultured in waste medium with the isolated strains and in a mixed culture with *L. plantarum*, using three culture variants. In subsequent stages, carotenoid powder was produced, and the carotenoid content, antioxidant capacity, and FTIR spectrum distribution were determined. The studies confirmed the possibility of using plant ferments as culture media. The extraction of powder enabled the concentration of carotenoids, obtaining the highest total fraction of carotenoids (TFC) for strains R-1 (2.85 mg/g d.w.) and R-2 (3.05 mg/g d.w.). FTIR spectra confirmed the presence of functional groups found in β-carotene standards in the resulting powders. At the same time, the obtained formulate exhibited bioactive properties by binding DPPH oxygen free radicals at a level of 66.80–78.05%.

## 1. Introduction

Carotenoids are 40-carbon terpenoids belonging to the isoprenoid compounds group. They are the most common natural pigments found in nature. Their main source is plants, which means they are phytochemicals with bioactive properties. Other sources of terpenoids include algae and microorganisms [1]. In nature, they are necessary for plant growth and photosynthesis. As biologically active pigments, they also have a beneficial effect on processes occurring in animal organisms [2]. Due to the presence of a series of coupled C=C bonds, they exhibit antioxidant properties, binding reactive oxygen species (ROS) and reactive nitrogen species (RNS). Therefore, carotenoids stimulate the immune system, supporting it in its response to apoptosis and mutagenesis processes [3]. In addition to their bioactive functions, carotenoids are used in the food industry as natural food additives due to their color. Accordingly, their properties are in line with the growing consumer awareness of proper nutrition. Currently, the production of synthetic carotenoids dominates the global market. This contributes to the generation of waste that is hazardous to the environment. Furthermore, the production of carotenoids using chemical synthesis often requires expensive raw materials, catalysts, and advanced equipment. The search for alternatives to the chemical synthesis of carotenoids has forced the market to calculate costs and estimate the profitability of producing natural compounds. Minimizing production costs could be achieved by utilizing waste. In addition to its pro-environmental effect, the use of organisms’ natural abilities to produce carotenoids promotes the development of new cultivation technologies. The use of post-production waste is a particularly interesting direction. It represents an opportunity for the innovative use of waste, which is an increasing problem in various industries. Waste from dairy, brewing, plant, and oil industries may be a rich source of nutrients, but for most carotenoid organisms, they are toxic or insufficient for the effective synthesis of the expected natural pigments [4]. Yeast is one of the few producers of these compounds exhibiting special adaptive properties to stressful environmental conditions. Their exploitation not only promotes waste utilization but also contributes to the development of ecological activities in line with the ‘zero waste’ motto, which allows natural carotenoids to compete with their synthetic analogues.

Research using plant-based waste mainly involves molasses, fruit, and vegetable waste (orange peels, sweet lemon peels, banana peels, mango peels, pea pods, and cauliflower/cabbage ends) [4,5]. The brewing industry also constitutes a source of waste, with researchers utilizing wasted grains as a source of water extracts and syrups for research purposes. In addition, research has also been conducted with coffee pulp extract, coffee bean husks, and tea waste [6]. Attempts to utilize industrial waste are associated not only with reducing the production costs of microbial carotenoids, but also with the desire to utilize the carbon and nitrogen compounds they contain. The use of plant post-ferments as a medium for yeast cultivation and carotenoid synthesis is an under-researched area [6]. The increasing popularity of fermented foods, including plant-based types, is expected to generate increasing amounts of post-production waste. This waste could be utilized by exploiting its components to produce bioactive compounds such as carotenoids [7]. An example of raw materials that are increasingly being subjected to fermentation are legumes and pseudocereals, including quinoa. Quinoa is used as a food or fodder plant. The fermentation process used by scientists increases the availability of complete protein and fiber contained in the seeds. However, standardizing the process of carotenoid production using waste requires a lot of analysis. One reason for this is the potentially variable composition of waste, which may affect the carotenoid fractions produced [4,8].

The aim of this article was to evaluate the possibility of using plant waste after quinoa fermentation to produce carotenoids using environmental yeasts and reference strains. In addition, the study determined the effect of co-cultivation with lactic acid bacteria on the intensity of carotenoid synthesis in three culture variants. Furthermore, FTIR spectrum analysis was performed and the antioxidant capacity of the compounds was determined for the most efficient variants.

## 2. Results

### 2.1. Yeast Strains

Environmental screening resulted in the isolation of a total of six strains fulfilling the selection criteria—three strains from the aquatic environment, two from lentils, and one from dairy products. Based on biochemical identification, it was determined that the isolated strains belonged to *R. mucilaginosa* (strains SR-20, SR-50, R-1 and R-2) and *R. glutinis* (strains SR-60 and M-66) (Table 1).

For the reference strains, information on the place of isolation was obtained from the strain characterization cards. Two isolates representing the species *R. mucilaginosa* and the strain *Sporobolomyces roseus*, also belonging to the *Sporidiobolales* family, were used.

### 2.2. Culture Media

Culture variant I used a standard laboratory medium with a carbon to nitrogen ratio of 2:1 for yeast culture. For plant post-ferment wastes (variants II and III), the C content expressed as reducing sugars was determined to be 3.44 ± 0.39 g/L. The nitrogen content was determined to be 0.3 g/L.

### 2.3. Carotenoid Production

Statistical analysis (ANOVA, *p* < 0.05) confirmed significant differences in dry cell weight (DCW) content for all strains in variant I. The highest biomass was obtained for strains SR-60 (5.69 g/L) and WUT-10 (5.27 g/L). The highest amounts of VCC were determined for strain SR-60 (2.03 mg/L), which correlated with the highest DCW. The total fraction of carotenoids (TFC) content differed statistically significantly for all strains except R-1 and R-2 (Table 2). However, the highest TFC amounts were determined for strains M-66 and SR-20 (Table 2), isolated from mozzarella and the digestive system of fish, respectively (Table 1).

The statistical analysis (ANOVA, *p* < 0.05) confirmed the significance of the differences for variant II in the obtained yeast biomass (DCW). The highest DCW values were determined for the *R. mucilaginosa* SR-20 strain and the WUT-167 reference strain. No statistically significant differences were found for the other isolates. The exception was the WUT-182 strain, for which no growth was observed in the culture medium. The DCW values determined for individual *Rhodotorula* strains do not correlate with the TFC. Statistically significant differences were found between TFC amounts. The highest amounts of carotenoids were detected in strain R-1, isolated from lentil seeds (597.07 μg/g d.w.). A similar level was determined for strains SR-50, WUT-10, and R-2 (Table 2).

In co-culture strains with *L. plantarum* (variant III), the highest DCW was determined for the reference strain WUT-167, while the lowest was determined for R-1, for which a statistical significance was confirmed. The highest TFC level (>400 μg/g d.w.) was extracted from cultures containing strains SR-50 and M-66. No statistically significant differences in TFC content were found for strains R-1 and WUT-167. As in variant II, in variant III, a correlation between VCC values and TFC content was confirmed. The exception was strain R-1 (Table 2).

The basic YPG laboratory medium (variant I) used for yeast cultivation was not favorable for the intensification of carotenoid synthesis, which may have been due to the low C:N ratio. The exception was strain 66, isolated from mozzarella, where 531.70 μg/g d.w. was determined on the YPG medium. However, the highest TFC, using post-fermentation waste as a medium (variant II), was isolated for strain R-1, i.e., 597.07 μg/g d.w. In most variants, higher TFC amounts were determined for variant II. The exception was strain M-66, where the use of co-culture (variant III) enabled an increase in TFC from 298.53 μg/g d.w. to 414.80 μg/g d.w. All strains showed growth in the culture variants used, apart from the reference strain WUT-182. The indicated strain showed the ability to form biomass and synthesise carotenoids only on YPG medium (variant I) (Table 2).

For subsequent analyses, strains exhibiting the highest ability to synthesise carotenoids on media using plant post-fermentation waste (variants II and III) were selected. Strains WUT-182, SR-20, and SR-60 were rejected. Analyses of the carotenoid content in the obtained powder showed significant differences compared to the results for the wet pellet mass. In the case of variant II, the highest TFC values were determined for strain R-2 (3.05 mg/g d.w). A similar value was also observed for strains R-1 and WUT-167, respectively, at 2.85 and 2.25 mg/g d.w. Other strains, i.e., SR-50, M-66, and WUT-10, exhibited TFC values below 2.0 mg/g d.w. However, the obtained results were not significantly different statistically, except those for strain M-66. The assessment of the effect of *L. plantarum* on the carotenoid yield of the tested strains proved that it depends on the strain. In co-culture cultures, strains R-1, R-2, and WUT-10 showed lower carotenoid productivity. In the remaining cultures, an increase in TFC was observed, with the highest values recorded for strains M-66 and SR-50 (Table 3).

The highest β-carotene content was found in the plant-derived strains R-1 and R-2 in variant II. Statistically significant differences in β-carotene content were found for strains WUT-167 and SR-50 in variant II, as opposed to the results for variant III. In variant III, lower variation in β-carotene content was observed. The highest β-carotene content in strains R-1 and R-2 correlated with the highest TFC values (Table 3).

**Figure 1:**
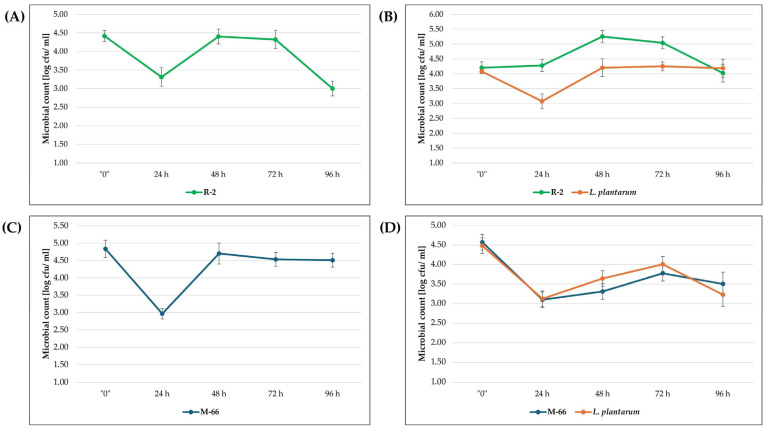
Biomass growth of the tested yeasts on waste substrate, depending on the variant used: variant II (**A**,**C**); variant III (**B**,**D**).

Biomass growth analysis was also performed for selected strains demonstrating the highest carotenoid productivity. All biomass growth diagrams show a stationary phase between 48–72 h. The log growth diagrams for strains R-1 and WUT-10 follow a similar pattern to that of strain R-2 (Figure 1A,B). However, strains WUT-167 and SR-50 follow a similar pattern to that of strain M-66 (Figure 1C,D). The biomass growth trends for yeast and *L. plantarum* were similar.

In the case of strain R-2, a higher abundance was observed than in *L. plantarum*, in contrast to the results for strain M-66. All presented cultures show a decrease in the number of microorganisms within the first 24 h after the start of cultivation, followed by an increase. The exception is the culture of strain R-2 in variant III (Figure 1B).

### 2.4. FTIR Analysis

**Figure 2:**
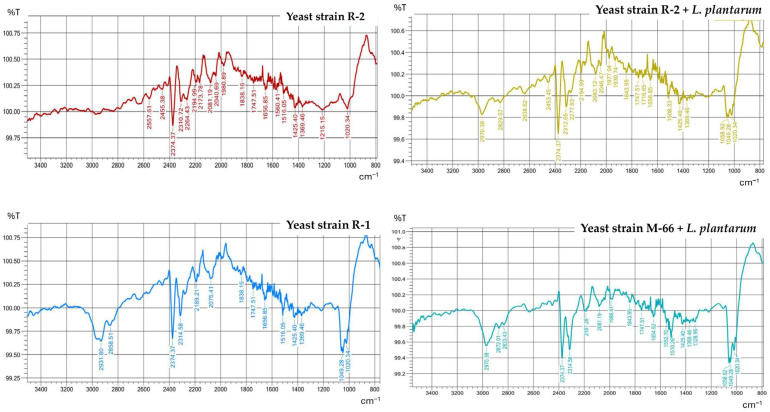
FTIR spectra of pigment extracted from the obtained powder.

FTIR spectra were obtained for the powder produced (Table 3). The separated absorption bands were recorded for wavelengths of approximately 1508, 1560, and 1654 cm^−1^, characteristic of β-carotene functional groups (Figure 2). In the case of powders obtained from strains SR-50, WUT-167, SR-1, and WUT-10, no effect of cultivation with *L. plantarum* (variant III) on the spectra was observed. The absence of absorption bands in the examined range was also observed (from 3500 cm^−1^ to 800 cm^−1^). However, the low TFC content of strain M-66 obtained in research variant II made it impossible to obtain an FTIR spectrum.

When comparing the powder spectra for the variants containing the highest TFC, i.e., R-2 and R-2, with *L. plantarum*, a clearer separation of absorption bands could be observed at wavelengths of 2000–2400 cm^−1^ (Figure 2).

### 2.5. DPPH Reduction Activity

The ability to reduce DPPH free radicals in cultures with *L. plantarum* (variant III) exhibited a higher reduction coefficient compared to the results for cultures with only yeasts (variant II). In monoculture cultures, the highest reduction ability was exhibited by powder obtained from strains R-2 and R-1. Cultures with *L. plantarum* of these strains increased the ability to reduce DPPH radicals. The same observation was made for strain SR-50, which showed an increase in activity from 67.68% to 84.15% in co-culture with *L. plantarum* (Table 4).

The correlation analysis of the examined parameters revealed no significant relationship between the DCW and TFC contents in culture variant II. However, a positive correlation was identified between TFC content in the powder and the ability to reduce DPPH radicals. This indicates a relationship between TFC increase and DPPH radical reduction ability (Table 5). However, a low correlation was found for the other variables.

A significant correlation between TFC powder and DPPH reduction was also observed for variant III of the culture in co-culture with *L. plantarum*. However, the correlation was negative. For the remaining variables, only weak correlations were observed, predominantly negative in direction (Table 6).

## 3. Discussion

### 3.1. Plant Waste as a Substrate for Carotenoid Synthesis

Currently, the production of carotenoids by yeast is not commercialized. The production cost, which includes both downstream and upstream processes, is one of the reasons for this. Alternative sources of carbon and nitrogen are being explored to reduce costs and enable the production of yeast biomass and their secondary metabolites [8]. Currently, the utilization of waste from various industries is being investigated. Scientists are utilizing both plant and animal waste. Plant waste includes beet juice, molasses, grape pomace, and potato waste. For plant post-fermentation waste, the literature only mentions brine obtained after radish fermentation [7]. However, there has been an increase in the use of fermentation processes for various raw materials, including quinoa. The main advantage of this process is the possibility of eliminating anti-nutritional components (saponins). In addition, quinoa contains complete protein, and the fermentation process can increase its digestibility [8]. The use of this waste as a culture medium requires analysis of its composition. One reason for this is the significant impact of changes in waste composition on the carotenoid fractions produced [9]. Yeast metabolism is mainly dependent on the availability and source of carbon. Glucose represents the simplest carbon substrate metabolized in the glycolytic pathway [10]. Therefore, one of the most important determinants of potential waste substrates is their sugar and nitrogen content, usually expressed as the C:N ratio. Accordingly, one of the most important determinants of potential waste substrates is their sugar and nitrogen content, usually expressed as the C:N ratio. These wastes may have different C:N ratios (5:1 to 45:1), depending on the source of the waste components and their possible modification. Literature reports indicate an optimal ratio of between 20:1 and 15:1. Šovljanski et al. (2025) [11] have used waste with a C:N ratio of 15:1 in the substrate. In the case of the post-fermentation waste used in this study, the C:N ratio was 12:1. The results obtained for the biomass formation of yeast strains (Table 2), except for *S. roseus*, indicate the possibility of using the obtained post-fermentation waste as a medium to produce carotenoids.

### 3.2. Carotenoid Production

The synthesis of carotenoids by yeast is determined by various physicochemical factors. Application of post-fermentation waste provided C and N compounds for biomass multiplication and carotenoid synthesis by the analyzed yeast strains. One of the reasons for this could be the low C:N ratio (2:1). Braunwald et al. (2013) [12] also demonstrated an increase in carotenoid synthesis with an increase in the C:N ratio. Low C content resulted in lower intensity of acetyl-CoA synthesis. In yeast, acetyl-CoA is converted in the mevalonate pathway to a key precursor molecule, i.e., isopentenyl pyrophosphate, enabling further carotenoid synthesis processes [9]. For the reference strains, the highest TFC levels (variant II) were obtained for the WUT-10 strain, which were twice as high as the TFC levels obtained for the *Rhodotorula* WUT-167 strain (Table 2). The only yeast representative that did not multiply in the waste medium was *S. roseus* (WUT-182) (Table 2). On the other hand, Marova et al. (2012) [13], using *S. roseus*, obtained yeast biomass growth on waste substrates containing a.o. potato extract. They obtained a DCW in the range of 4.61–5.25 g/L and a β-carotene content of 594.42–2780.00 μg/g d.w. in this research. Lack of growth of the WUT-182 strain may have been related to the poor nutrient content of the waste substrate or the strain’s low adaptability to growth under unfavorable environmental conditions. The environmental strains SR-20 and SR-60 exhibited the lowest TFC content. The reason for this phenomenon could be the origin of the strains from environments low in phytochemicals that inhibit yeast growth (saponins and phenolic compounds) [8]. The highest TFC concentrations were recorded in yeasts isolated from lentil grains, i.e., R-1, R-2, and strain SR-50, whose natural habitat was bottom sediments (Table 1). Similar TFC levels were reported by Ghilardi et al. (2020) [4], who observed comparable amounts of TFC when culturing on olive waste substrate. As for the previous results, no correlation was found between TFC and DCW content (Table 5).

An increase in carotenoid productivity can be obtained by applying stress factors, including high salinity or low/high pH. Malisorn et al. (2008) [7] demonstrated an increase in carotenoid and DCW production with increasing concentrations of brine used as a culture medium. In contrast, Kulczyk-Małysa & Bogusławska-Wąs (2024) [14] proved that pH 3 had no inhibition effect on carotenoid synthesis. The increase in carotenoid production also includes the co-culture of yeast with other microorganisms. Several studies have reported that carotenoid production increases in mixed cultures of carotenoids and microorganisms that do not exhibit the ability to produce carotenoids [15]. Zhang et al. (2019) [16] used *R. glutinis* and *Chlorella vulgaris* in starch wastewater. However, the cultivation of *R. rubra* and *Lactobacillus casei* [17] and *R. rubra* and yogurt starter in dairy industry waste also proved to be an effective combination [18]. In this research, the use of *L. plantarum* and selected strains (variant III) affected TFC growth only in the case of strain M-66. In the remaining culture variants, a decrease in TFC was observed compared to the results for the single culture—variant II (Table 2). Similar amounts of carotenoids in the research variants (II and III) were determined for strains SR-50 and R-2. This research demonstrates that carotenoid production by carotenoid-producing microorganisms increases as a result of interaction with other bacteria. Bacteria promote the utilization of nutrients in the medium that are unavailable to carotenoid-producing microorganisms [18,19,20], as illustrated by the logarithmic growth curves B and D (Figure 1). However, this property depends on the strain and results from possible competition for metabolic precursors, which can lead to the induction or inhibition of carotenogenesis [21]. In the case of strain R-2, co-culture with *L. plantarum* did not significantly increase carotenoid synthesis despite higher amounts of yeast biomass, which may have been due to competition (Figure 1B). The opposite situation was observed for strain M-66. The dominance of *L. plantarum* in the culture may have been a stress factor promoting the induction of carotenogenesis (Figure 1D). Therefore, it may be concluded that the used post-fermentation waste contained nutrients available for the growth of yeast isolates. However, co-cultivation caused no significant increase in carotenoid synthesis in most of the tested variants.

For the practical use of natural carotenoids obtained through microbial synthesis, the product obtained is converted into a dry form—powder. Moreover, the preparations must be made in the form that allows them to be applied during the technological process. The most used methods are conventional drying, spray drying, and freeze-drying. Obtaining carotenoids in powder form simplifies their application in various industries [22]. During this research, conventional drying was used. As a result, a higher TFC content was obtained in the biomass powder compared to that in the wet mass. The highest TFC values were indicated for strains R-1 and R-2 in monoculture (variant II). These strains are of plant origin, which may have improved their adaptation to the waste substrate used and may have resulted in higher TFC values. The analysis of the β-carotene content (Table 3) also indicates that strains R-1 and R-2 show the highest production of this fraction. Marova et al. (2012) [13] obtained a β-carotene content at the level of 594.42–2780.00 μg/g d.w. In the present research, results within the indicated range were recorded for strains of plant origin (R-1, R-2). A positive effect on TFC content in co-culture (variant III) was demonstrated for strains SR-50 and M-66 (Table 3). In most variants, the TFC content in both mono- and co-culture was not significantly different. The use of conventional drying may have adversely affected the structure of the carotenoids, inducing their degradation due to the temperature and duration of the process (50 °C/1 h). The presence of lactic acid fermentation biomass and the compounds it contains may have provided protection during drying. Consequently, no significant differences in TFC content were observed in the powder. The use of conventional drying could not achieve such high carotenoid production yields as those obtained using spray drying in the work of Bhosale et al. (2003) [22]. Carotenoids have a saturated structure and are therefore susceptible to isomerization and oxidation processes. Compared to the results for isomerization reactions, the products of oxidation are more numerous. There are several types of oxidation: auto-oxidation, enzyme-catalyzed oxidation, light-induced oxidation, and temperature-induced oxidation, which was a critical parameter in these studies [23]. Therefore, future studies will focus on selecting a high-efficiency biomass drying method to minimize the oxidation of carotenoid compounds.

### 3.3. FTIR Analysis

FTIR spectroscopy is based on the vibrational excitation of bonds by the absorption of infrared light energy. Functional groups may be associated with a characteristic infrared absorption band, resulting in their fundamental vibrations. In this study, FTIR spectra revealed the presence of various functional groups in the obtained powders. Most functional groups were detected in all carotenoids extracted from the powders, proving their similarity in chemical structure. All powder spectra (Figure 2) were dominated by three major maxima at 1508 cm^−1^, 1560 cm^−1^, and 1654 cm^−1^. The absorption bands around 1500–2000 cm^−1^ correspond to the double bound C=C stretching vibrations of β-carotene [24,25]. Also, according to Saha et al. [26], the maximum at 1635 cm^−1^, which is similar to absorption band 1654 cm-1 found in our study, represents C=C stretching. The maximum at 1654 cm^−1^ represented a C=O band, typical for a conjugated ketone present in carotenoid capsanthin [27]. Additionally, in the powder from R-1 and M-66 from *L. plantarum*, an absorption band at 2931 cm^−1^ and 2970 cm^−1^ was pronounced, indicating the presence of asymmetric stretching modes of C-H groups [24]. Similar results were obtained by Anshi et al. (2025) [28], observing a peak at a wavelength of 2916 cm^−1^. The absorption bands around 2374 cm^−1^ are due to the O=C=O stretching of carbon dioxide [24]. Carbon dioxide as a component of atmosphere is present in the powders, and despite the procedure of subtracting the background spectrum, the CO_2_ signals are present. The absorption band at 1049 cm^−1^ is close to the 1052 cm^−1^ band noted by Abubakar et al. (2025) [29] and attributed to the rocking vibrations of the (RH) C=C (RH) groups of carotenoids. The characteristic absorption bands for the β-carotene standard, according to Abubakar et al. (2025) [29], include three major absorption bands at 2922 (C−H), 1725 (C=O), and 959 cm^−1^ (C−C). According to Saha et al. (2015) [26], the absorption bands confirming the identification of carotenoids occur at 965 (bending C=C), 1385 (bending C-H), 1635 (stretching C=C), and 2920 (bending C-H) cm^−1^ (Figure 2).

### 3.4. Antioxidant Potential

Reactive oxygen species (ROS) and oxygen free radicals are often considered to be factors determining the occurrence of adverse changes in the human body. The accumulation of harmful forms of oxygen generates oxidative stress. An imbalance between the production of free radicals and the presence of antioxidants leads to damage to cell components, including the nucleic acids. Therefore, oxygen free radicals are often associated with the occurrence of mutagenesis and carcinogenesis [30]. The prevention of these processes involves the supply of natural antioxidants, which also include carotenoids. In this research, the obtained carotenoid powders were tested for their DPPH reduction potential. It was demonstrated that all samples, regardless of the cultivation variant, had the ability to reduce DPPH radicals in the range of 66.80–84.15% (Table 4). The highest reduction values were observed for compounds obtained from strains R-1 and R-2 (70.16–78.05%), of plant origin, and strain SR-50 (84.15%), isolated from the natural environment. All the strains indicated represented the species *R. mucilaginosa* (Table 1). Similar results were obtained by Moreira et al. (2018) [6] using solid coffee waste as a culture medium. Subsequently, Banerjee et al. (2025) [31] demonstrated the highest antioxidant activity in the *Rhodotorula* sp. KSB1 strain at 72.00%. The antioxidant activity of yeast carotenoids proves that pigments in microorganism cells have a protective function against induced oxidative damage [32]. In the present research, a positive correlation was also demonstrated between carotenoid content and DPPH reduction capacity for cultures with yeast alone (variant II) as opposed to the results for co-culture (variant III) (Table 5 and Table 6). Saha et al. (2015) [26] also confirmed an increase in DPPH reduction with increasing carotenoid concentration in the pigment extracted from *Sporidiobolus pararoseus*.

## 4. Materials and Methods

### 4.1. Yeast Strains

The research used standard strains of carotenoid yeasts *Rhodotorula mucialginosa* WUT-167 and WUT-10, *Sporobolomyces roseus* WUT-182 (collection of the Warsaw University of Technology, Faculty of Chemistry, Warsaw, Poland), and environmental isolates: SR-20, SR-50, SR-60, R-1, R-1, and M-66, isolated from dairy and plant products, water, and bottom sediments of natural water bodies and fish. The selection of yeast isolates was based on colony color, ranging from pink to red, which indicated the potential for carotenoid compound production. The selection and identification of the isolated yeast strains was performed according to previous research [14].

### 4.2. Plant Waste Used

The plant post-ferment was obtained as a waste product from the fermentation of quinoa grains, which was performed at 5% *v*/*v* (6.0 × 10^5^ cfu/mL) using *Lactobacillus plantarum* (archive of the Department of Applied Microbiology and Human Nutrition Physiology, West Pomeranian University of Technology, Szczecin, Poland). Fermentation was performed for 2 days at 30 °C. After fermentation, the grains were separated from the liquid. The obtained post-ferment was sterilized at 121 °C for 15 min, cooled, and stored at 4 °C until it was used.

To determine the C/N ratio in the post-ferment, the total number of reducing sugars was determined using the NDS acid method according to Łopusiewicz et al. (2019) [33], with our own modification. The nitrogen content was determined using the Kjeldahl method in accordance with the AOAC [34].

### 4.3. Yeast Culture Methods

Selected and standard yeast strains with a constant inoculum of 6.0 × 10^5^ cfu/mL were used in the experiment. The cultures were maintained in 100 mL flasks with 50 mL of culture medium, shaken continuously at 120 rpm, and exposed to light at 25 °C for 5 days [35]. Three culture variants were tested: (I) YPG medium (2% glucose, 1% peptone, 1% yeast extract) with a C:N ratio of 2:1 and selected yeast strains; (II) plant post-ferment (100%) with a C:N ratio of 12:1 and selected yeast strains; (III) plant post-ferment (100%) with a C:N ratio of 12:1 and selected yeast strains and *L. plantarum* in a 1:1 ratio.

### 4.4. Extraction and Determination of Carotenoid Content

The extraction of carotenoids synthesized by yeast strains was performed according to the methods of Kot et al. 2023 [36], with our own modification [14]. To determine the total carotenoid content, the absorbance of the extracts was measured using a BioSpectrometer basic spectrophotometer (Eppendorf, Hamburg, Germany) at wavelengths of 500 nm for TFC and 450 nm for β-caroten [14,37]. Volumetric carotenoids concentration (VCC) was expressed in mg/L of culture medium, and total fraction of carotenoids was expressed in μg/g d.w. (TFC) was calculated according to the methods of Chen et al., 2006 [38] using the following formula:

TFC = VCC/DCW

where:

VCC—volumetric carotenoids concentration (mg/L);

DCW—dry cell weight (g/L);

TFC—total fraction of carotenoids (in μg/g d.w.).

The dry cell weight (DCW) was determined in falcon tubes, which had been dried for 2 days at 50 °C. After weighing the falcon tubes, 10 mL of each culture was added and centrifuged at 8000 rpm for 10 min. The supernatant was decanted, and the biomass in the falcon tubes was dried at 50 °C. The falcon tubes were weighed every 24 h until there were no significant differences in weight. The results have been presented in grams of dry mass per liter of medium (g/L) [38].

Biomass growth diagrams were determined using the drop-plate method according to the work of Herigstad et al. (2001) [39], with our own modification. The research covered the determination of *L. plantarum* on MRS medium (OXOID, Basingstoke, UK) and the analyzed yeast on Sabouraud Dextrose Agar medium (OXOID, Basingstoke, UK). Incubation was carried out at 30 °C/72 h and 25 °C/5 days for *L. plantarum* and yeast, respectively. Determinations were made on the day the culture was established and for the next 96 h, with samples taken every 12 h. The number of colonies obtained from the culture were counted and reported in log cfu/mL.

### 4.5. Powder Preparation

The culture was centrifuged at 8000 rpm for 10 min to obtain the powder. The supernatant was decanted, and the pellet was suspended in sterile distilled water in a 1:1 ratio to the wet weight of the pellet. The suspension obtained in this way was dried at 50 °C for one hour. The dried pellet was mechanically crushed to obtain powder. The powder was stored in tightly closed Eppendorf tubes without access to light at room temperature.

### 4.6. Determination of DPPH Radical Reduction Ability

The total antioxidant activity was determined based on the reduction of 1,1-diphenyl-2-picrylhydrazyl (DPPH) (TCI, Tokyo, Japan). The reactions were performed in a DPPH solution in 100% methanol (7.4 mg/100 mL) diluted with distilled water in a 1:10 ratio. The test sample was added in a 1:5 ratio and left (without access to light) at 23 °C for 30 min. After this time, the absorbance was measured at λ = 517 nm [31,40]. The result was given as % antioxidant activity according to the following formula:

% inhibition = 100 (A_0_ − A_av_.)/A_0_

where:

A_av_.—average absorbance value of the tested solution containing the antioxidant;

A_0_—absorbance of the DPPH radical solution.

### 4.7. FTIR Analysis

FTIR spectra of the samples were obtained at room temperature using an IRTracer-100 Fourier transform infrared spectrophotometer with QATR 10 (Shimadzu, Kyoto, Japan). The samples were scanned in the range from 3500 cm^−1^ to 800 cm^−1^ at a resolution of 4.0. The numbers of scans of the sample was 20.

### 4.8. Statistical Analysis

All experiments were repeated three times, and the data are presented as mean ± standard deviation (SD). Statistical significance was determined using analysis of variance (one-way ANOVA), followed by Tukey’s test. The compared values were considered significantly different at *p* < 0.05.

Correlations between the variables of the strain used, the co-occurrence of yeast with *L. plantarum*, DCW, TFC powder, and DPPH reduction were assessed using Pearson’s correlation coefficient (r) at a significance level of *p* < 0.05. All analyses were performed using Statistica software version 13.03 (StatSoft Polska, Krakow, Poland).

## 5. Conclusions

The presented research demonstrated the possibility of synthesizing bioactive carotenoids on a waste substrate, i.e., plant post-ferment. Furthermore, it creates another area of unused potential for fermented product waste, therefore minimizing the production costs of carotenoids of microbial origin.

The research enabled the acquisition of a collection of carotenoid yeasts from various natural environments and from processed products. At the same time, all isolates, except for the representative of the genus *Sporobolomyces*, were shown to be able to produce carotenoids. The highest TFC values in monoculture were determined for strains R-1, R-2, and SR-50. In contrast, coculture enabled the highest TFC values to be obtained for SR-50 and M-66. This confirmed the influence of the natural environment of yeast on their ability for adaptation to waste substrates and carotenoid production.

The extraction of powder enabled the concentration of carotenoids, obtaining the highest TFC for strains R-1 (2.85 mg/g d.w.) and R-2 (3.05 mg/g d.w.). The highest amounts of β-carotene were also obtained from strains R-1 and R-2 (0.65 mg/g d.w.). However, no significant effect of cultivation with *L. plantarum* was observed, except for in strain 66, which originated from a dairy product. FTIR spectra confirmed the presence of functional groups found in β-carotene standards in the resulting powders. At the same time, the obtained formulant exhibited bioactive properties by binding DPPH oxygen free radicals at a level of 66.80–78.05%.

Further studies will focus on increasing the TFC content in the powder by selecting an appropriate drying method, along with its parameterization.

## Figures and Tables

**Table 1:** Strains used in research.

Strain Designation	Strain Species	Place of Isolation
	Natural Environment	
SR-20	*Rhodotorula mucilaginosa*	Fish digestive system
SR-50	*Rhodotorula mucilaginosa*	Bottom sediments
SR-60	*Rhodotorula glutinis*	Water from a water reservoir
Plants		
R-1	*Rhodotorula mucilaginosa*	Lentil grains
R-2	*Rhodotorula mucilaginosa*	Lentil grains
WUT-167	*Rhodotorula mucilaginosa*	Rowan tree
WUT-182	*Sporobolomyces roseus*	Quince fruit
	Dairy Products	
M-66	*Rhodotorula glutinis*	Mozzarella
WUT-10	*Rhodotorula mucilaginosa*	Kefir

**Table 2:** Impact of the research variant on the analyzed parameters.

Yeast Strain	DCW	VCC	TFC
	g/L	mg/L	μg/g d.w.
Variant I			
SR-20	2.42 ± 0.20 ^e^	1.05 ± 0.02 ^bc^	433.90 ± 7.81 ^b^
SR-50	3.92 ± 0.12 ^c^	1.10 ± 0.02 ^b^	281.30 ± 6.80 ^d^
SR-60	5.69 ± 0.10 ^a^	2.03 ± 0.35 ^a^	357.20 ± 3.51 ^c^
R-1	1.81 ± 0.23 ^f^	0.11 ± 0.01 ^f^	62.20 ± 1.33 ^h^
R-2	1.19 ± 0.57 ^h^	0.07 ± 0.02 ^f^	56.70 ± 1.62 ^h^
WUT-167	3.10 ± 0.14 ^d^	0.56 ± 0.05 ^d^	180.56 ± 5.08 ^f^
WUT-182	1.58 ± 0.09 ^g^	0.31 ± 0.03 ^e^	195.15 ± 2.51 ^e^
M-66	1.89 ± 0.67 ^f^	1.01 ± 0.21 ^c^	531.70 ± 7.03 ^a^
WUT-10	5.27 ± 1.12 ^b^	0.55 ± 0.03 ^d^	99.62 ± 2.89 ^g^
Variant II			
SR-20	2.30 ± 0.36 ^ab^	0.18 ± 0.04 ^bc^	79.77 ± 2.66 ^e^
SR-50	1.53 ± 0.35 ^bc^	0.70 ± 0.17 ^a^	452.27 ± 8.63 ^b^
SR-60	1.49 ± 0.17 ^c^	0.27 ± 0.03 ^b^	181.17 ± 3.55 ^de^
R-1	1.21 ± 0.29 ^c^	0.71 ± 0.07 ^a^	597.07 ± 9.25 ^a^
R-2	1.49 ± 0.22 ^c^	0.61 ± 0.14 ^a^	403.63 ± 7.52 ^bc^
WUT-167	2.57 ± 0.33 ^a^	0.58 ± 0.07 ^a^	227.23 ± 10.99 ^cd^
WUT-182	0.00 ± 0.00 ^d^	0.00 ± 0.00 ^c^	0.00 ± 0.00 ^f^
M-66	1.69 ± 0.40 ^bc^	0.51 ± 0.13 ^ab^	298.53 ± 10.55 ^c^
WUT-10	1.20 ± 0.18 ^c^	0.56 ± 0.03 ^a^	472.13 ± 5.85 ^b^
Variant III			
SR-20	1.29 ± 0.12 ^cd^	0.07 ± 0.01 ^ef^	29.50 ± 2.21 ^e^
SR-50	2.23 ± 0.10 ^ab^	0.95 ± 0.04 ^a^	426.80 ± 4.59 ^a^
SR-60	1.64 ± 0.19 ^bcd^	0.10 ± 0.03 ^ef^	40.61± 3.19 ^e^
R-1	1.19 ± 0.17 ^d^	0.30 ± 0.05 ^de^	253.07 ± 4.82 ^c^
R-2	1.98 ± 0.61 ^abc^	0.69 ± 0.26 ^b^	349.37 ± 8.99 ^b^
WUT-167	2.60 ± 0.16 ^a^	0.60 ± 0.07 ^bc^	229.93 ± 2.59 ^c^
WUT-182	0.00 ± 0.00 ^e^	0.00 ± 0.00 ^f^	0.00 ± 0.00 ^f^
M-66	1.41 ± 0.18 ^cd^	0.58 ± 0.08 ^bc^	414.80 ± 8.41 ^a^
WUT-10	2.16 ± 0.23 ^ab^	0.37 ± 0.03 ^cd^	171.73 ± 8.45 ^d^

±standard deviation; ^a, b, c, d, e, f, g, h^—column difference significance at *p* < 0.05; DCW—dry cell weight; VCC—volumetric carotenoids concentration; TFC—total fraction of carotenoids.

**Table 3:** Total carotenoid content in powder.

Yeast Strain	Variant II	Variant III
	TFC [mg/g d.w.]	
SR-50	1.55 ± 0.10 ^c^	2.60 ± 0.04 ^a^
R-1	2.85 ± 0.04 ^a^	2.24 ± 0.09 ^b^
R-2	3.05 ± 0.08 ^a^	2.33 ± 0.01 ^b^
WUT-167	2.25 ± 0.01 ^b^	2.58 ± 0.04 ^ab^
M-66	0.97 ± 0.02 ^d^	2.00 ± 0.06 ^c^
WUT-10	1.40 ± 0.04 ^c^	1.26 ± 0.02 ^d^
β—carotene [mg/g d.w.]		
SR-50	0.34 ± 0.01 ^c^	0.49 ± 0.01 ^ab^
R-1	0.65 ± 0.02 ^a^	0.55 ± 0.03 ^a^
R-2	0.65 ± 0.02 ^a^	0.45 ± 0.02 ^b^
WUT-167	0.46 ± 0.01 ^b^	0.49 ± 0.02 ^ab^
M-66	0.18 ± 0.05 ^d^	0.42 ± 0.01 ^b^
WUT-10	0.21 ± 0.01 ^d^	0.18 ± 0.05 ^c^

±standard deviation; ^a, b, c, d^—column difference significance at *p* < 0.05; TFC—total fraction of carotenoids.

**Table 4:** DPPH reduction activity.

Yeast Strain	Variant II	Variant III
	DPPH [%]	
SR-50	67.68 ± 1.61 ^cd^	84.15 ± 0.61 ^a^
R-1	71.14 ± 0.35 ^b^	70.16 ± 3.15 ^c^
R-2	76.83 ± 1.61 ^a^	78.05 ± 2.19 ^b^
WUT-167	69.92 ± 0.35 ^bc^	69.11 ± 0.93 ^c^
M-66	66.87 ±0.70 ^d^	69.90 ± 0.61 ^c^
WUT-10	66.80 ± 0.93 ^d^	70.12 ± 0.62 ^c^

±standard deviation; ^a, b, c, d^—column difference significance at *p* < 0.05.

**Table 5:** Correlation between selected variables for variant II culture.

Variable	Yeast Strain	DCW [g/L]	TFC in Powder [mg/g d.w.]	DPPH [%]
**Yeast strain**	1.00	0.09	0.30	0.24
**DCW [g/L]**	0.09	1.00	-	−0.01
**TFC in powder [mg/g d.w.]**	0.30	-	1.00	0.91 *

* difference significance at *p* < 0.05; DCW—dry cell weight; TFC—total fraction of carotenoids.

**Table 6:** Correlation between selected variables for variant III culture.

Variable	Yeast Strain	DCW [g/L]	TFC in Powder [mg/g d.w.]	DPPH [%]
**Yeast strain**	1.00	−0.25	−0.47	−0.85 *
**DCW [g/L]**	−0.25	1.00	0.18	0.27
**TFC in powder [mg/g d.w.]**	−0.47	0.18	1.00	0.46

* difference significance at *p* < 0.05; DCW—dry cell weight; TFC—total fraction of carotenoids.

## Data Availability

No new data were generated or analysed in this study. Therefore, data sharing is not applicable.
